# Supplementary material for: Prognostic biomarkers for predicting papillary thyroid carcinoma patients at high risk using nine genes of apoptotic pathway
Source: PLoS One. 2021 Nov 12;16(11):e0259534. doi: 10.1371/journal.pone.0259534 (PMC8589158; doi:10.1371/journal.pone.0259534)
Supplement: S2 File — (DOCX) [file pone.0259534.s002.docx]

**S2 File**

**
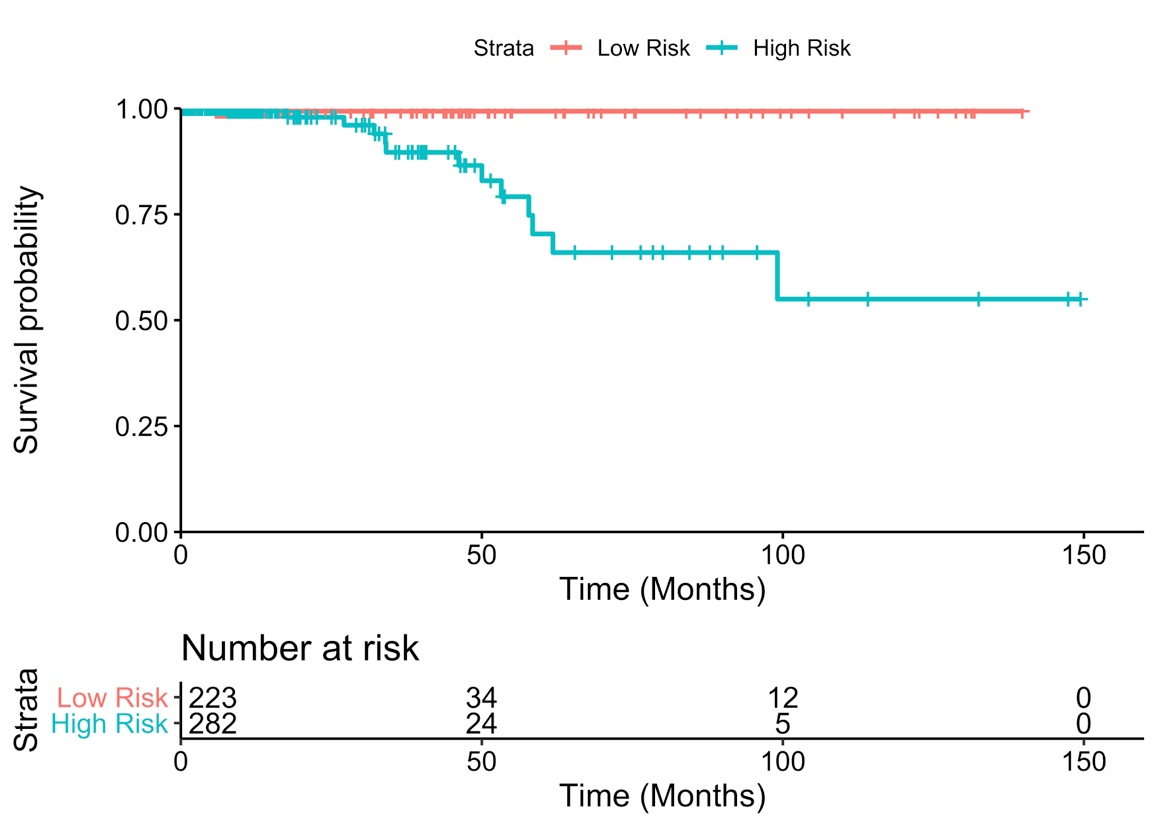
**

**Fig 1** Risk stratification of PTC patients using prognostic index (PI) model. Patients with PI> -3.29x10^5^ (estimated using cutp() package in R) were found to be at higher risk with HR=17.55, p=5.88x10^-3^, C=0.65, %95CI 2.29-134.72 and logrank-p=6.73x10^-5^.


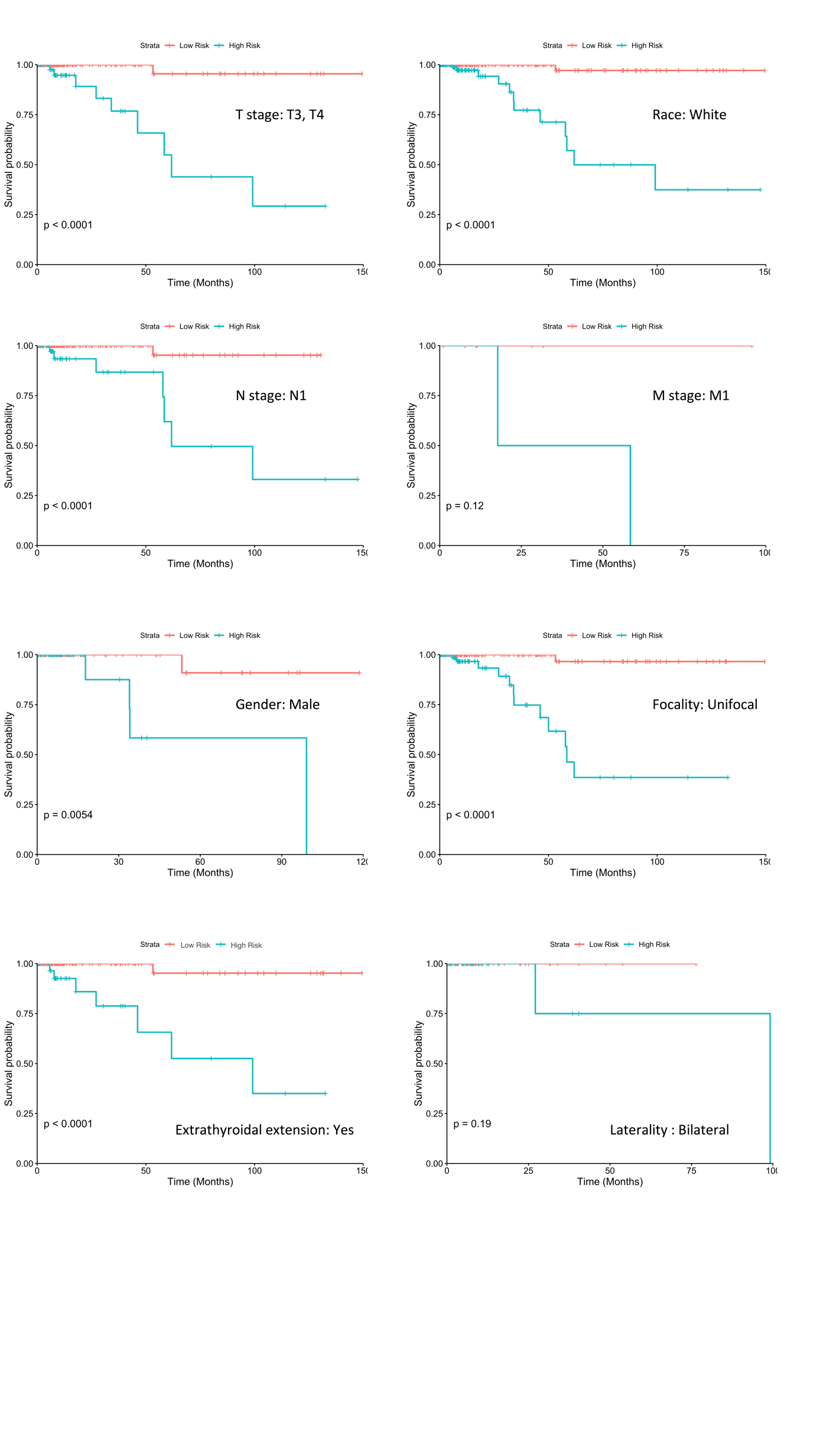


**Fig 2** Sub-stratification of clinico-pathological high risk groups by 9-gene voting model. Logrank p values show significant segregation between survival curves.
