# Supplementary material for: Prognostic biomarkers for predicting papillary thyroid carcinoma patients at high risk using nine genes of apoptotic pathway
Source: PLoS One. 2021 Nov 12;16(11):e0259534. doi: 10.1371/journal.pone.0259534 (PMC8589158; doi:10.1371/journal.pone.0259534)
Supplement: Attachment — Submitted filename: Response to Reviewers.docx [file pone.0259534.s003.docx]

**Response to Reviewers**

The authors are thankful to the Editor and the Reviewers for investing time and effort into reviewing this manuscript. The suggested comments are appreciated and were taken into consideration in the revised version of the manuscript as detailed below.

**Journal requirements**

1. Please ensure that your manuscript meets PLOS ONE's style requirements, including those for file naming. The PLOS ONE style templates can be found at <https://journals.plos.org/plosone/s/file?id=wjVg/PLOSOne_formatting_sample_main_body.pdf> and <https://journals.plos.org/plosone/s/file?id=ba62/PLOSOne_formatting_sample_title_authors_affiliations.pdf>.

**Response**: We have re-formatted our manuscript as per the style requirements provided. The file names have also been changed accordingly.

2. In order to ensure your study is reproducible, please provide the TCGA project dataset name(s) included in your study, and/or state the specific inclusion criteria used to select patient samples and the dates of your search.

**Response**: We have now added the TCGA project name along-with the date of access in the ‘Materials and methods’ section.

3. Thank you for stating the following in the Acknowledgments Section of your manuscript:
“Authors are thankful to Indraprastha Institute of Information Technology (IIIT-Delhi) and University Grants Commission (UGC, India) for financial support and fellowships.”

We note that you have provided funding information that is not currently declared in your Funding Statement. However, funding information should not appear in the Acknowledgments section or other areas of your manuscript. We will only publish funding information present in the Funding Statement section of the online submission form.
Please remove any funding-related text from the manuscript and let us know how you would like to update your Funding Statement. Currently, your Funding Statement reads as follows:
“The author(s) received no specific funding for this work.”

Please include your amended statements within your cover letter; we will change the online submission form on your behalf.

**Response**: We have removed the “Acknowledgements” section from the manuscript. The funding statement remains the same as earlier.

4. We note that Figure 7 in your submission contain copyrighted images. All PLOS content is published under the Creative Commons Attribution License (CC BY 4.0), which means that the manuscript, images, and Supporting Information files will be freely available online, and any third party is permitted to access, download, copy, distribute, and use these materials in any way, even commercially, with proper attribution. For more information, see

our copyright guidelines: <http://journals.plos.org/plosone/s/licenses-and-copyright>.
 
We require you to either (1) present written permission from the copyright holder to publish these figures specifically under the CC BY 4.0 license, or (2) remove the figures from your submission:
 
a. You may seek permission from the original copyright holder of Figure 7 to publish the content specifically under the CC BY 4.0 license.
 
We recommend that you contact the original copyright holder with the Content Permission Form (<http://journals.plos.org/plosone/s/file?id=7c09/content-permission-form.pdf>) and the following text:
“I request permission for the open-access journal PLOS ONE to publish XXX under the Creative Commons Attribution License (CCAL) CC BY 4.0 (<http://creativecommons.org/licenses/by/4.0/>). Please be aware that this license allows unrestricted use and distribution, even commercially, by third parties. Please reply and provide explicit written permission to publish XXX under a CC BY license and complete the attached form.”
 
Please upload the completed Content Permission Form or other proof of granted permissions as an "Other" file with your submission.
 
In the figure caption of the copyrighted figure, please include the following text: “Reprinted from [ref] under a CC BY license, with permission from [name of publisher], original copyright [original copyright year].”
 
b. If you are unable to obtain permission from the original copyright holder to publish these figures under the CC BY 4.0 license or if the copyright holder’s requirements are incompatible with the CC BY 4.0 license, please either i) remove the figure or ii) supply a replacement figure that complies with the CC BY 4.0 license. Please check copyright information on all replacement figures and update the figure caption with source information. If applicable, please specify in the figure caption text when a figure is similar but not identical to the original image and is therefore for illustrative purposes only.

**Response**: We understand the copyright policy of PLOS ONE. After getting comments from the journal, we again read the license conditions of “The Human Protein Atlas” (source of images). Please see link <https://www.proteinatlas.org/about/licence>, wherein they have mentioned the license information that “The Human Protein Atlas is licensed under the [Creative Commons Attribution-ShareAlike 3.0 International License](http://creativecommons.org/licenses/by-sa/3.0/) for all [copyrightable parts](http://sciencecommons.org/data/dbfaq) of database”. We carefully followed the instructions mentioned on “The Human Protein Atlas” and provided appropriate citations to these images.

Please review your reference list to ensure that it is complete and correct. If you have cited papers that have been retracted, please include the rationale for doing so in the manuscript text, or remove these references and replace them with relevant current references. Any changes to the reference list should be mentioned in the rebuttal letter that accompanies your revised manuscript. If you need to cite a retracted article, indicate the article’s retracted status in the References list and also include a citation and full reference for the retraction notice.

**Response**: We have reviewed the reference list as suggested.

**Additional Editor Comments**

As such the study is interesting and well-studied. Both the reviewers recommended for publication. Authors are asked to address the reviewer comments.

**Response:** We are thankful to the editor as well as both the reviewers.

**Reviewers' comments**

**Reviewer #1**: Manuscript entitled “Prognostic biomarkers for predicting papillary thyroid carcinoma patients at high risk using nine genes of apoptotic pathway” is a well-written manuscript with novelty. Authors have selected the various methods to obtain the data. The methods they used are appropriate and the data they presented show a clear support of their conclusion. Manuscript can be acceptable after proof reading for minor typos.

**Response**: We thank the reviewer for recommending our work. As suggested, we have re-checked the manuscript for typos and made the corrections.

**Reviewer #2**: This is an interesting study and you have collected good datasets. The paper is well written and structured.
Line 1: Aberrant expression of apoptotic genes has been associated with papillary thyroid carcinoma. Have been is correct.
Line 28: Can you detail the the sentence, risk prediction models?
Line 86: Apoptosis is the process for eliminating cells in multicellular organisms? Can you add on additional points?

**Response:** We appreciate the provided comments**.** We have now made the suggested correction and provided additional details for Line 28 and Line 86.
