# Supplementary material for: Prognostic biomarkers for predicting papillary thyroid carcinoma patients at high risk using nine genes of apoptotic pathway
Source: PLoS One. 2021 Nov 12;16(11):e0259534. doi: 10.1371/journal.pone.0259534 (PMC8589158; doi:10.1371/journal.pone.0259534)
Supplement: Attachment — Submitted filename: Response to Reviewers.docx [file pone.0259534.s004.docx]

**Response to Reviewers**

The authors are thankful to the Editor for providing relevant suggestions. The suggested comments were taken into consideration in the revised version of the manuscript as detailed below.

**Journal requirements**

1. Please review your reference list to ensure that it is complete and correct. If you have cited papers that have been retracted, please include the rationale for doing so in the manuscript text, or remove these references and replace them with relevant current references. Any changes to the reference list should be mentioned in the rebuttal letter that accompanies your revised manuscript. If you need to cite a retracted article, indicate the article’s retracted status in the References list and also include a citation and full reference for the retraction notice.

**Response**: The references were reviewed using Pubmed. Only the following two articles were unavailable on PubMed but are NOT retracted:

(a) Wang SH, Baker JR. Apoptosis in thyroid cancer. Thyroid Cancer (Second Edition): A Comprehensive Guide to Clinical Management. Humana Press; 2006. pp. 55–61. doi:10.1007/978-1-59259-995-0_6

Book chapter available at <https://link.springer.com/chapter/10.1007/978-1-59259-995-0_6>

(b) Pedregosa F, Varoquaux G, Gramfort A, Michel V, Thirion B, Grisel O, et al. Scikit-Learn: Machine Learning in Python. J Mach Learn Res. 2011;12: 2825–2830.

Article available at <https://www.jmlr.org/papers/v12/pedregosa11a.html>

If the problem persists, we request you to provide us with the titles of the references, which seem to be an issue.

**Additional Editor Comments**

1. Change the following line 33-35 ---It can be categorized into four major subtypes: i) papillary thyroid carcinoma (PTC), ii) follicular thyroid carcinoma (FTC), iii) medullary thyroid carcinoma (MTC), and 35 iv) anaplastic thyroid carcinoma (ATC).--- as exactly rewritten below and do not include MTC.

"Thyroid cancer developed from follicular cells can be mainly categorized into papillary (PTC), follicular (FTC), and anaplastic thyroid cancer (ATC)."

**Response:** Thank you. We have re-phrased the sentence as suggested by the Editor.
